# Method-Comparison Validation of a Novel Capillary Blood Collection Kit, True Dose^®^ TD-EPI, for Therapeutic Drug Monitoring of Epirubicin

**DOI:** 10.3390/ph19020226

**Published:** 2026-01-28

**Authors:** Serena De Chiara, Nektarios Komninos, Oscar P. B. Wiklander, Per Rydberg, Elham Hedayati

**Affiliations:** 1 True Dose AB, 171 65 Solna, Sweden; serena.dechiara@truedose.se (S.D.C.);; 2 Unit for Biomolecular and Cellular Medicine, Department of Laboratory Medicine, Karolinska Institute, 171 77 Stockholm, Sweden; 3 Breast Center, Karolinska, Comprehensive Cancer Center, Karolinska University Hospital, 171 76 Solna, Sweden; 4 Department of Oncology-Pathology, Karolinska Institute, 171 77 Solna, Sweden; 5 Department of Oncology, South General Hospital, 118 83 Stockholm, Sweden

**Keywords:** capillary blood sampling, anthracyclines, epirubicin, breast neoplasms/drug therapy, blood specimen collection/methods, mass spectrometry/methods

## Abstract

**Background:** Therapeutic drug monitoring (TDM) is a promising strategy to personalize chemotherapy dosing, especially for agents with narrow therapeutic indices such as epirubicin. However, widespread adoption is hindered by logistical challenges associated with venous blood sampling and centralized laboratory workflows. **Objective:** This study aimed to perform a method-comparison validation of the True Dose^®^ TD-EPI microsampling kit by verifying analytical agreement between capillary and venous epirubicin measurements in real patient samples. The study focuses on analytical performance and does not constitute validation of the whole decentralized workflow, including unsupervised patient self-sampling. **Methods:** 13 patients with early-stage breast cancer receiving the first cycle of neoadjuvant or adjuvant epirubicin were enrolled. Capillary samples were collected using the finalized TD-EPI kit (Cap-TD) at 2.5 h (*n* = 13) and/or 48 h (*n* = 10) post-infusion and stored at room temperature for 72 h before analysis. Matched venous samples were analyzed using both conventional protein precipitation (“Traditional”) and a modified lab-based True Dose workflow (Lab-TD). Epirubicin concentrations were quantified via validated liquid chromatography–tandem mass spectrometry (LC–MS/MS). **Results:** Cap-TD concentrations showed strong agreement with Traditional venous values (r = 0.953), with minimal bias (mean difference = 0.013 μM) in Bland–Altman analysis. Passing–Bablok regression confirmed analytical equivalence. Intra-assay variability remained within ICH M10 guidelines (CV ≤ 15%), and recovery was unaffected by 72 h ambient storage. Lab-TD results closely matched Traditional workflows, supporting reproducibility. **Conclusions:** The TD-EPI kit enables accurate decentralized monitoring of epirubicin, eliminating the need for venous access, cold-chain logistics, or in-clinic sampling. These findings support its integration into personalized oncology care and future applications in home-based TDM. Trial Registration: This study is part of an approved protocol registered in the EU Clinical Trials Register (EUCT Number 2024-514818-12-00; EudraCT Number 2017-000641-44; registration date: 15 June 2017).

## 1. Introduction

Despite decades of clinical use, dosing of cytotoxic chemotherapy remains imprecise [1]. Most regimens, including anthracycline-based epirubicin, rely on fixed body surface area (BSA)-based dosing, a strategy that overlooks key patient-specific variables, such as hepatic and renal function, comorbidities, and pharmacogenetic variability [1,2]. Consequently, systemic drug exposure can vary significantly between patients receiving the same nominal dose. This variability contributes to a narrow therapeutic index: underdosing may reduce efficacy, while overdosing increases toxicity, often without real-time confirmation of exposure [1,2].

Therapeutic drug monitoring (TDM) is widely adopted in fields such as psychiatry and infectious diseases to individualize dosing and control toxicity [3,4,5,6]. Despite established exposure–response and exposure–toxicity relationships for many anticancer drugs, TDM remains underutilized in oncology [7,8,9,10,11,12]. The main barrier is operational. Venous sampling, the gold standard for TDM, is logistically challenging to coordinate, as accurate pharmacokinetic (PK) assessment after short-infusion chemotherapies such as epirubicin requires blood sampling within short, precisely defined post-infusion time windows [13]. Constraints such as limited clinic capacity, staffing shortages, and patient burden further hinder timely sample collection [14,15,16], making integration into routine workflows challenging. This leads to missed opportunities for individualized dose optimization.

Capillary microsampling presents a promising alternative. This approach enables low-volume blood collection through a minimally invasive method that can be performed outside clinical settings. It facilitates sampling at late PK time points, which are often missed in standard workflows. Previous studies have shown that microsampling can improve TDM feasibility, reduce logistical barriers, and preserve analyte integrity under ambient storage [15,17,18]. However, current microsampling devices face limitations, including haematocrit sensitivity, inconsistent blood volume collection, and reliance on user technique [19,20,21,22]. Most also lack integrated internal standards (IS), increasing variability during self-sampling.

To address these limitations, the True Dose^®^ platform was developed as a fully integrated capillary microsampling system designed for TDM of oncology agents. The TD-EPI kit, an epirubicin-specific version of the platform, incorporates fixed-volume sampling, pre-filled stabilization solvent, and automated IS release to improve analytical consistency and enable ambient-temperature transport.

This study aimed to perform a method-comparison validation of the True Dose^®^ TD-EPI microsampling kit by verifying analytical agreement between capillary and venous epirubicin concentrations at two clinically informative time points: 2.5 h (peak distribution) and 48 h (late elimination) post-infusion. The study focuses on analytical performance and does not constitute validation of the whole decentralized workflow, including unsupervised patient self-sampling. The primary objective was to assess analytical equivalence between Cap-TD and traditional venous sampling using pre-defined Bland–Altman and Passing–Bablok criteria. Secondary objectives included: (i) evaluating intra-method reproducibility of venous samples processed via the modified True Dose^®^ workflow (Lab-TD) or the conventional method (Traditional), and (ii) assessing operational robustness of TD-EPI under 72 h ambient storage conditions.

Together, these outcomes are intended to establish the reliability, reproducibility, and practical suitability of the TD-EPI kit for decentralized TDM. Successful method comparison may help overcome key logistical barriers to implementing personalized chemotherapy dosing in routine clinical practice.

## 2. Results

### 2.1. Patient Cohort and Sample Availability

Thirteen female patients receiving epirubicin during their first chemotherapy cycle were enrolled between 16 December 2024 and 10 June 2025. All 13 participants provided matched capillary and venous samples at the early PK time point (2.5 h, C1S1), whereas 10 participants contributed samples at the late time point (48 h, C1S2) due to temporary constraints on the mobile nurse’s availability for home-based collections.

Across both time points, a total of 23 capillary samples, 69 Lab-TD venous replicates, and 69 Traditional venous replicates were analyzed.

### 2.2. Analytical Equivalence Between Capillary and Venous Samples

#### Individual-Level Agreement

Epirubicin concentrations obtained from capillary microsampling (Cap-TD) were compared against those from the Traditional venous workflow at both PK time points. Epirubicin concentrations obtained from Cap-TD closely matched those from the Traditional workflow, with a mean Cap-TD/Traditional ratio of 1.14, indicating that capillary-derived epirubicin concentrations were, on average, 14% higher than those obtained from venous sampling (Table 1. Two samples, P4S1 and P10S2 (Table 1), deviated significantly (−31% and −28.9%, respectively) due to known procedural issues (e.g., low volume or prolonged sampling time). Intra-assay precision for the Lab-TD and Traditional workflows was consistently ≤15% CV across replicates.

All paired measurements, including those associated with documented pre-analytical deviations, were retained in the Passing–Bablok and Bland–Altman analyses. One sample (P4, C1S1) lay outside the limits of agreement, while another (P10, C1S2) was close but within the limits; including these observations did not affect regression parameters, mean bias, or the limits of agreement.

### 2.3. Passing-Bablok Analysis

**Figure 1:**
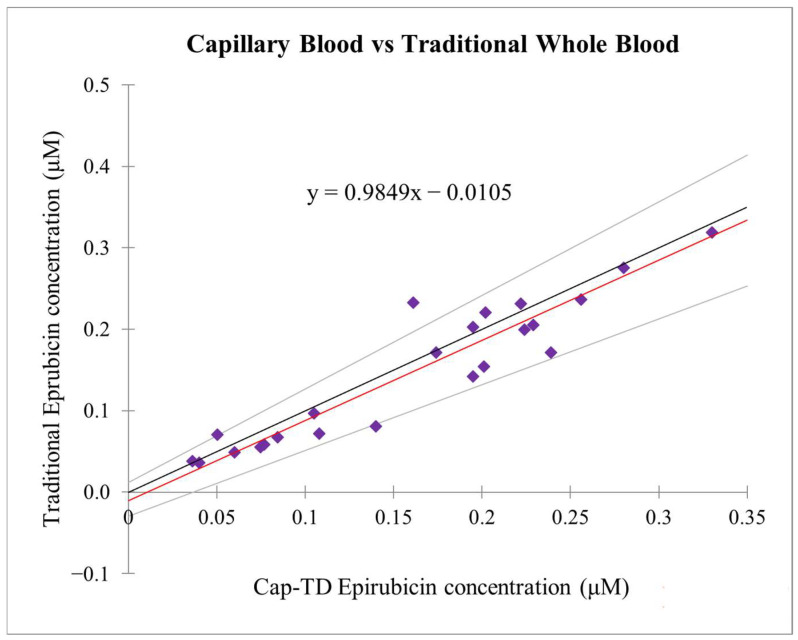
Method comparison of epirubicin concentrations: capillary True Dose^®^ (Cap-TD) vs. Traditional venous sampling (Passing–Bablok). Purple symbols represent individual paired measurements; the solid red line indicates the Passing-Bablok regression, and the flanking lines denote the 95% confidence intervals.

Passing–Bablok regression confirmed analytical equivalence, with no evidence of systematic or proportional bias. The slope (0.985; 95% CI: 0.915–1.042) was close to 1, and the intercept (0.0105; 95% CI: −0.022–0.034) met pre-defined equivalence criteria, indicating strong agreement between capillary and venous measurements without systematic over- or underestimation (Figure 1). Nearly all data points fell within the 95% confidence band, with one outlier (P4S1) linked to insufficient capillary volume.

Across all patient samples collected at the early (S1, 2.5 h post-infusion) and late (S2, 48 h post-infusion) PK time points, Cap-TD concentrations showed a strong correlation with Traditional venous measurements (Pearson r = 0.953), indicating a robust linear relationship.

Bootstrap resampling (5000 iterations) was used to estimate 95% confidence intervals for the slope and intercept, providing more robust uncertainty estimates. The bootstrap CI for the slope ranged from 0.891 to 1.167, and the intercept CI ranged from −0.0046 to 0.0405 µM, both meeting analytical equivalence criteria.

Leave-one-patient-out sensitivity analyses showed that exclusion of any individual patient, including outlier P4S1, did not materially alter regression estimates (Supplementary Figure S2).

### 2.4. Bland–Altman Analysis

**Figure 2:**
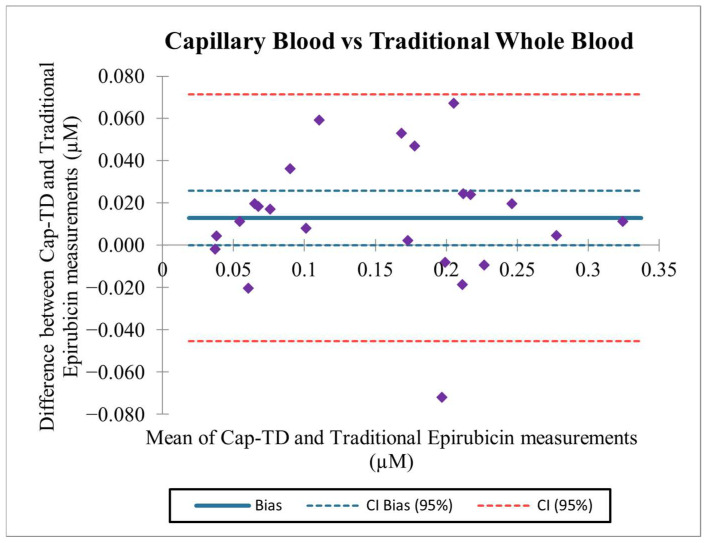
Agreement analysis of epirubicin concentrations: capillary True Dose^®^ (Cap-TD) vs. Traditional venous sampling (Bland–Altman). Purple symbols represent individual paired measurements.

Bland–Altman analysis showed a mean bias of 0.013 μM, with the 95% confidence interval (−0.045 to 0.071 µM) including zero, indicating no systematic over- or underestimation by Cap-TD relative to Traditional (Figure 2). The majority of paired differences (22 out of 23) fell within the 95% limits of agreement, and no evidence of proportional bias was observed across the concentration range.

A Bland–Altman analysis was also performed on relative percent differences, yielding a mean bias of 14.1% and limits of agreement of −33.8% to 62.0%.

Bootstrap confidence intervals for the absolute bias ranged from 0.0006 to 0.0246 µM. In contrast, the bootstrap CI for the percent bias was 4.3% to 24.3%, supporting stability of the agreement despite higher dispersion at low concentrations.

Normality of the difference distributions was confirmed using the Shapiro–Wilk test (*p* > 0.05 for both absolute and percent differences), indicating no significant deviation from normality.

In addition, linear regression of the paired differences against the average of the two methods showed no proportional bias (slope = −0.013, *p* = 0.87).

On average, Cap-TD workflow concentrations were 14.4% higher than those from the Traditional workflow. However, this slight positive bias (≈0.01 µM) was within the acceptable agreement limits and not clinically significant (Figure 1). Slightly wider limits of agreement at the later sampling time point (S2) were observed, consistent with increased relative variability at lower epirubicin concentrations. The most significant deviation (P4S1) was documented during sample collection.

Leave-one-out and leave-one-patient-out sensitivity analyses confirmed robustness of the Bland–Altman estimates to outlier exclusion (Supplementary Figure S1).

Together, these findings suggest that, on average, capillary readings are slightly higher than venous readings, but the discrepancy is minor, and the two techniques provide essentially the same information. In practice, either method could be used interchangeably, acknowledging the small positive bias (≈0.01 µM) when interpreting absolute values.

### 2.5. Cross-Method Concentration Overview

**Figure 3:**
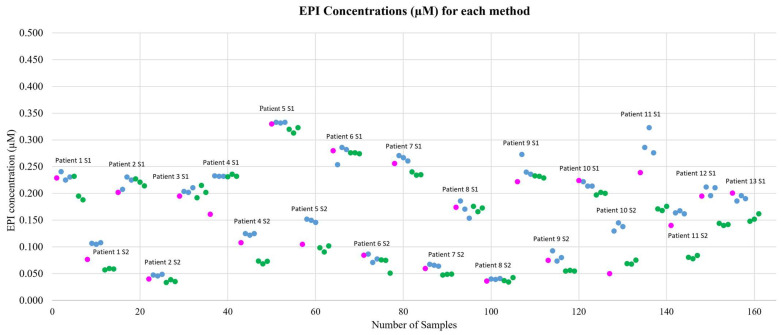
Epirubicin (EPI) concentrations (µM) by method: capillary (Cap-TD; pink), venous processed using the True Dose workflow (Lab-TD; blue), and conventional venous method (Traditional; green). Each point represents an individual sample. Data shown for two time points: S1 (2.5 h) and S2 (48 h) post-infusion. Absolute epirubicin concentrations are shown to preserve clinical interpretability across time points. Relative differences between methods are reported in Table 1 (as ratios and CV%) and analyzed in Figure 2 (Bland–Altman analysis).

Epirubicin concentrations measured by capillary (Cap-TD) closely align with both venous methods (Lab-TD and Traditional) across patients and time points, supporting cross-method agreement and analytical reproducibility (Figure 3 and Table S2 in the Supplementary File, which includes individual raw area under the curve (AUC) and concentration values).

### 2.6. Reproducibility of Venous Replicates

Intra-assay reproducibility was assessed using CV% across triplicate venous replicates. The Lab-TD replicates demonstrated consistently high precision (CV: 0.17–11.8%), while Traditional replicates showed broader variability (CV: 0.4–21%), particularly at lower concentrations (e.g., P6S2).

Capillary samples were collected as single replicates and not formally assessed for precision. Deviations from expected concentrations were limited to samples with known collection issues, such as reduced blood volume or prolonged sampling time (e.g., P4S1, P4S2, P5S2, P10S2). However, deviations were infrequent and primarily associated with procedural errors, reinforcing the importance of proper user training and sample handling in decentralized TDM workflows.

## 3. Discussion

This method-comparison study validated the True Dose^®^ TD-EPI capillary microsampling kit for TDM of epirubicin in early-stage breast cancer. Capillary-derived concentrations showed strong agreement with standard venous workflows, meeting pre-defined criteria for analytical equivalence using Passing–Bablok regression, Bland–Altman analysis, and a robust Pearson correlation (r = 0.953). Together, these results confirm the method’s reliability and support its use for decentralized PK sampling in oncology settings.

Analytical and operational robustness were demonstrated under real-world conditions. Capillary samples remained stable after 72 h of ambient storage, simulating decentralized transport logistics. While this method-comparison validation focused on a 72-h window, our earlier proof-of-concept study demonstrated that TD-EPI prototypes remained stable for up to 14 days under ambient conditions, with higher measured concentrations than those observed with traditional workflows, likely reflecting more complete matrix equilibration and solvent extraction [13,23]. Epirubicin concentrations remained within ±15% of baseline values, with analytical precision improving over time due to delayed matrix equilibration and extraction kinetics. Building on prior work, this study assessed the finalized TD-EPI kit (v4.0) in routine clinical conditions, demonstrating reproducible results across two clinically relevant post-infusion time points. The observed Cap-TD-to-Traditional concentration ratio of 1.14 aligns with previously reported recovery patterns [7]. The LC–MS/MS method met ICH M10 and EMA validation standards, with intra-assay variability for venous replicates within acceptable limits (CV ≤ 15%). Moreover, venous samples processed using the TD workflow (Lab-TD) showed lower variability than those processed with Traditional lab methods, reinforcing the kit’s analytical robustness. Only one isolated outlier (P4S1) was detected, with no evidence of systematic bias or analyte degradation. These findings confirm that the TD-EPI kit can yield clinically actionable PK data without relying on venous access or cold-chain infrastructure.

Epirubicin remains a cornerstone of anthracycline-based chemotherapy regimens, yet exhibits marked inter-individual PK variability (clearance CV% ≈ 25–30%) [2]. Because BSA-based dosing explains <10% of this variability [1], many patients receive suboptimal exposure, risking either preventable toxicity or reduced efficacy. This PK unpredictability is compounded by narrow therapeutic windows and the absence of established plasma concentration thresholds to guide real-time dosing. Although exposure–toxicity relationships are well documented [24], TDM for anthracyclines remains underused, partly due to the logistical burdens of venous sampling, timed collection, and laboratory constraints. TDM for anthracyclines remains underused, partly due to logistical burdens of venous sampling, timed collection, and laboratory constraints. Our findings address this unmet need by validating a whole-blood capillary microsampling platform designed explicitly for anthracycline TDM, enabling precise, home-based PK assessment at clinically relevant timepoints (2.5 h and 48 h post-infusion) without cold-chain transport.

Patient recruitment was limited by contemporary breast cancer treatment practices in Sweden. Due to national mammography screening, earlier-stage diagnosis without the need for chemotherapy, and increasing adoption of non-anthracycline regimens, the number of patients receiving epirubicin has declined substantially. Consequently, the pool of eligible patients with earlier-stage breast cancer for paired pharmacokinetic sampling is limited, particularly within a single-center prospective study over a defined time period. Despite this constraint, paired capillary and venous samples were obtained at pharmacokinetically informative time points from the majority of enrolled participants, which constitutes the critical requirement for method-comparison validation.

Bland–Altman analysis showed capillary concentrations were, on average, 14.4% higher than venous measurements (mean difference ≈ 0.01 µM). This slight positive bias is unlikely to be clinically significant, particularly since epirubicin dosing is not guided by fixed plasma thresholds. However, if capillary sampling is adopted for routine dose adjustment, future protocols may require defining capillary-specific therapeutic ranges.

These results align with recent international initiatives advocating precision dosing in oncology, including the U.S. Food and Drug Administration’s (FDA) *Project Optimus* [25,26,27,28] and The *Patient-Centered Dosing Initiative* [29], both of which emphasize exposure–response characterization and the integration of TDM. Furthermore, large-scale meta-analytic data confirm the ongoing clinical relevance of epirubicin in dose-dense neo-/adjuvant regimens, reinforcing the importance of individualized exposure optimization in modern chemotherapy [30].

Our study advances the field by validating a capillary-based workflow tailored to intravenous cytotoxic chemotherapy. Using a fully integrated, ambient-stable epirubicin kit, we demonstrated that fixed-volume sampling combined with automated IS release yields robust analytical agreement with conventional venous workflows. This supports the clinical applicability of the True Dose^®^ platform for decentralized TDM. Additionally, we compared venous samples processed with the same True Dose^®^ protocol (Lab-TD) to those analyzed via traditional methods. The strong concordance between these workflows suggests that the True Dose^®^ sample preparation process, with built-in IS and simplified protein precipitation, offers a reliable, interchangeable solution for clinical laboratories operating LC–MS/MS systems.

By enabling decentralized capillary sampling and ambient transport, the TD-EPI kit addresses key barriers to broader TDM adoption. Fixed-volume collection, automated IS release, and pre-filled stabilizing solvent minimize user-dependent variability, making it feasible for home or outpatient workflows. Compared to other microsampling approaches, such as dried blood spots (DBS), volumetric absorptive microsampling (VAMS), or saliva collection, liquid-based capillary sampling may offer advantages in volume control, matrix handling, and analytical consistency [18,21]. However, no direct comparison was performed in this study, and future work should explore relative performance across platforms.

Our findings also underscore the importance of tracking variability in drug exposure. Inter-patient differences were most evident at the 48-h post-infusion time point, when drug levels were lower and more sensitive to pre-analytical influences. This pattern mirrors earlier observations for cytotoxic agents such as docetaxel [31], further supporting the clinical relevance of late-phase TDM for optimizing dosing and minimizing toxicity.

The TD-EPI kit aligns with broader healthcare policy and regulatory trends. As van der Kleij et al. note, centralized TDM workflows often face logistical challenges, particularly in rural or outpatient settings [15]. The TD-EPI kit addresses these by enabling ambient-stable, decentralized sampling that integrates with standard LC–MS/MS workflows. Its compatibility with existing lab infrastructure supports efforts across the EU to expand equitable access, reduce dependence on external supply chains, and promote regional biomanufacturing sovereignty.

This study has limitations; firstly, capillary samples were collected by trained mobile nurses rather than by patients themselves. While this ensured protocol adherence, it does not fully reflect unsupervised self-sampling conditions. However, previous usability testing of earlier TD-EPI prototypes demonstrated over 90% success in self-sampling [32]. As the kit design remains unchanged, a follow-up study is planned to evaluate real-world self-sampling performance. Second, while two key PK time points were assessed, longitudinal sampling across multiple treatment cycles would better capture inter-occasion variability. Third, the cohort included only women with early-stage breast cancer, which limits generalizability to other tumor types, genders, and age groups. Fourth, although hematocrit was not assessed in this cohort, previous work using the TD-EPI system demonstrated minimal impact of Hct on epirubicin recovery. Nonetheless, future validation studies should incorporate hematocrit measurement and stratified analyses to confirm robustness across diverse clinical populations [33]. Finally, this study is limited by its modest sample size and real-world sampling constraints, which restrict the precision of agreement estimates and preclude definitive claims of analytical equivalence. However, the use of non-parametric bootstrap confidence intervals and leave-one-patient-out sensitivity analyses mitigates the influence of individual patients and repeated measurements. The observed stability of agreement metrics supports the robustness of the findings. Overall, these results demonstrate the agreement and feasibility of TD-capillary sampling relative to traditional venous sampling under clinical conditions.

To be clinically valuable, TDM must offer timely results. The TD-EPI kit’s ambient stability enables compatibility with postal workflows and centralized lab analysis. With overnight shipping and routine LC–MS/MS processing, dosing recommendations could be returned in time to inform the next chemotherapy cycle, typically scheduled every 2–3 weeks, without disrupting standard care.

To expand its utility, future work should validate the True Dose^®^ platform across broader populations, including older adults, patients with comorbidities, and other treatment modalities such as oral targeted agents or biologics. Further studies are also needed to confirm the feasibility of unsupervised self-sampling in routine settings. Moreover, should capillary sampling be adopted for routine TDM or dose-adjustment in future protocols, it may become necessary to define capillary-specific reference or target ranges. Ultimately, integration with adaptive dosing algorithms, including AI-enabled model-informed precision dosing, may allow real-time personalization of cancer therapy.

These findings support the growing shift toward decentralized, patient-centered oncology care. Regulatory bodies such as the EMA increasingly endorse microsampling in both clinical trials and routine care. Platforms like True Dose^®^ help operationalize this vision, offering a scalable, validated approach to TDM that enhances access, supports health system efficiency, and advances the promise of precision medicine.

## 4. Materials and Methods

### 4.1. Study Design

This prospective validation was conducted as part of the ongoing single-center TailorDose II study at the Karolinska University Hospital Breast Centre in Stockholm, Sweden. Eligible participants were adults with early-stage breast cancer scheduled to receive their first cycle of epirubicin-based chemotherapy in a neoadjuvant or adjuvant setting. Written informed consent was obtained from all participants prior to any study procedures.

This primary aim was to evaluate analytical equivalence between capillary and venous workflows for epirubicin quantification, comparing the True Dose^®^ TD-EPI kit (Cap-TD) with venous samples processed via a modified True Dose workflow (Lab-TD) or a standard clinical reference workflow (Traditional). Paired samples were collected at two pharmacokinetically informative time points (2.5 h and 48 h post-infusion), selected to capture both peak distribution and late elimination phases under real-world clinical conditions. The three workflows, Cap-TD (decentralized capillary sampling), Lab-TD (lab-controlled analogue), and Traditional (standard clinical method), were assessed in parallel. The three workflows were designed to isolate the impact of sample type and handling on epirubicin measurement (Table 2).

### 4.2. Sampling Procedures

Matched venous and capillary samples were obtained during the first chemotherapy cycle. The first sampling time point (Cycle 1, Sample 1; C1S1) occurred 2.5 h post-infusion and was collected in the clinic by a research nurse. The second sampling time point (Cycle 1, Sample 2; C1S2) occurred 48 h post-infusion and was collected during a home visit by a mobile research nurse.

### 4.3. Capillary Sampling (Cap-TD)

Capillary blood (50 µL) was collected using the True Dose^®^ TD-EPI microsampling kit. Blood was collected using a fixed-volume Minivette^®^ (50 µL) and dispensed directly into a TD-kit tube pre-loaded with a stabilization solvent mixture consisting of 1 mL isopropanol/methanol (1:1) with 0.1% formic acid. Closing the cap activated the integrated internal-standard matrix and stainless-steel bead, releasing IS into the solvent and allowing homogenization of the blood sample. Samples were manually shaken by the user for 30 s and stored at room temperature for 72 h to simulate real-world ambient conditions for transport and storage; however, the shaking force or frequency was not standardized. This was evaluated during product development, and it was concluded that with 30 s shaking, results are consistent regardless of shaking frequency.

### 4.4. Venous Sampling

Venous blood (5 mL in Ethylenediaminetetraacetic acid (EDTA) blood tube) was collected at each time point via routine phlebotomy and processed using two analytical workflows, Traditional and Lab-TD, each performed in triplicate to assess reproducibility.

### 4.5. Traditional Reference Workflow

Three venous aliquots (50 µL each) were transferred into tubes pre-loaded with 1 mL isopropanol/methanol 1:1 containing 0.1% formic acid, along with 20 µL of doxorubicin IS (1 µM). Samples were vortexed for 30 s and immediately stored at −26 °C for 72 h before analysis.

### 4.6. True Dose Venous Workflow (Lab-TD)

Three additional venous aliquots (50 µL each) were pipetted into activated True Dose^®^ TD-EPI kits. Firstly, the cap was closed to release IS and an integrated stainless-steel bead; blood was then added with an automatic pipette, and the tubes were manually shaken for 30 s. Samples were then stored for 72 h at room temperature to simulate ambient transport as Cap-TD samples.

Therefore, each participant contributed one Cap-TD sample and six venous replicates (three Lab-TD and three Traditional) at each PK time point.

### 4.7. Sample Processing

Following the 72 h storage period, all sample types underwent identical preparation to ensure analytical comparability. Tubes were sonicated in an ice-cooled bath for 10 min, centrifuged at 9000 rpm for 10 min, and 400 µL of supernatant was transferred to autosampler vials and stored at −26 °C until liquid chromatography–tandem mass spectrometry (LC–MS/MS) analysis.

### 4.8. Bioanalysis

All samples were analyzed using a validated LC–MS/MS method compliant with International Council for Harmonisation (ICH) M10 and European Medicines Agency (EMA) guidelines [34].

### 4.9. LC–MS/MS Method for Epirubicin Quantification

Epirubicin concentrations were quantified using a validated LC–MS/MS method on a Waters Xevo TQ-S micro triple-quadrupole mass spectrometer coupled with an Acquity UPLC system (Waters Corp, Milford, MA, USA). Chromatographic separation was performed using a Waters CSH C18 column (100 × 2.1 mm, 1.7 µm) with a mobile phase gradient consisting of:

Mobile phase A: 0.1% formic acid in water

Mobile phase B: 100% acetonitrile with 0.1% formic acid

The gradient increased mobile phase B from 0% to 28% at 3.80 min, then to 95% between 3.90 and 4.40 min, followed by column reconditioning.

Flow rate was 0.6 mL/min, with an injection volume of 2 µL.

Detection was conducted in positive electrospray ionization (ESI^+^) mode using multiple reaction monitoring (MRM). Transitions monitored were:

Epirubicin: *m/z* 544 → 130 (cone voltage: 35 V; collision energy: 15 V)

Internal standard:

Doxorubicin: *m/z* 544 → 130 (cone voltage: 35 V; collision energy: 15 V).

Daunorubicin was only used as an auxiliary reference during method development to mitigate local ion-suppression effects (later elution) and as an internal-standard integrity indicator via a stable daunorubicin/doxorubicin ratio (ratio increases upon doxorubicin degradation).

### 4.10. Method Validation

The development and validation of the LC–MS/MS analytical method for the quantification of epirubicin in capillary and venous whole blood samples were conducted in accordance with EMA guidelines and the ICH M10 bioanalytical method validation guideline [34]. To evaluate the analytical method and ensure reliability of the generated data, the following validation parameters were assessed: specificity, selectivity, linearity, lower limit of quantification (LLOQ), accuracy, precision, and carry-over.

Stability was assessed for TD-kits, which remained stable for up to 14 days at room temperature after blood collection. Selectivity and specificity were evaluated by analyzing drug-free whole blood samples processed using the TD-EPI workflow, including both double blanks (without IS and Blank IS (with IS added)). Selectivity was considered acceptable when interfering signals did not exceed 20% of the analyte response at the LLOQ and 5% of the IS response.

Calibration curves and quality control (QC) samples were prepared by spiking fresh human venous blood with known concentrations of epirubicin. The LLOQ was 0.02 µM, and the Upper Limit of Quantification (ULOQ) was 2 µM. The calibration curve was prepared fresh on the day of analysis. The QC samples at three concentration levels (low, medium, and high) were used to assess within-run and between-run accuracy and precision. Accuracy was expressed as the percent deviation from the nominal concentration, and precision as the coefficient of variation (CV%). Acceptance criteria required accuracy and precision to remain within ±15% for all QC levels, and within ±20% at the LLOQ, in line with ICH M10 recommendations [34]. Carry-over was evaluated by injecting high-concentration samples followed by blank samples. Carry-over was considered negligible when residual analyte and IS signals in the blank did not exceed 20% and 5% of the LLOQ response, respectively. Quality control sample performance of the runs is included in the Supplementary File (Table S1).

### 4.11. Reagents and Reference Standards

Epirubicin hydrochloride, doxorubicin hydrochloride, and daunorubicin hydrochloride reference standards were obtained from the British Pharmacopeia and Sigma-Aldrich (St. Louis, MO, USA). Solvents for extraction and chromatographic separation, including methanol, isopropanol, acetonitrile, and formic acid, were purchased from Sigma-Aldrich. Ultrapure water was generated using a Milli-Q system, Merck Millipore (Darmstadt, Germany).

### 4.12. Consumables and True Dose Kit Components

**Figure 4:**
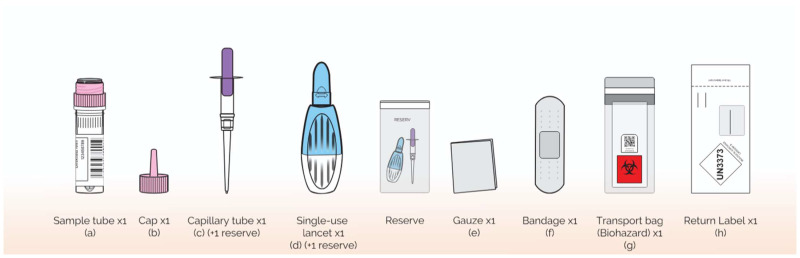
Consumables and Components of the True Dose^®^ TD-EPI kit: (**a**) True Dose^®^ microtube with integrated internal standard (IS) matrix; (**b**) Activation pin; (**c**) Sarstedt (Numbrecht, Germany) Minivette^®^ Capillary EDTA, 50 µL + 1 reserve; (**d**) BD (Dublin, Ireland) Microtainer^®^ safety lancet (2.0 mm) + 1 reserve; (**e**) Non-woven gauze; (**f**) Adhesive plaster; (**g**) Biohazard collection bag; (**h**) Prepaid return envelope with UN3373-compliant label.

The True Dose^®^ TD-EPI kit contains a polypropylene microtube pre-filled with extraction solvent and a cap-integrated IS. The kit is designed for self-sampling blood collection using the included safety lancet and fixed-volume capillary device (Figure 4).

### 4.13. Endpoints and Statistical Analysis

The primary endpoint was to assess analytical equivalence between epirubicin concentrations in capillary samples processed with the True Dose^®^ TD-EPI kit (Cap-TD) and venous samples processed using the Traditional workflow. This study was designed as a method-comparison validation study and was not powered for formal hypothesis testing; therefore, no a priori sample size or power calculation was performed.

Analytical equivalence was evaluated using complementary statistical methods: Passing–Bablok regression to assess bias, and Bland–Altman analysis to assess agreement limits. Equivalence was concluded if the 95% confidence interval (CI) for the slope included 1.00 (no proportional bias) and the CI for the intercept included 0 (no constant bias). Bland–Altman analysis was used to assess systematic bias and Limits of Agreement (LoA). Equivalence required the 95% CI for the mean bias (±1.96 × SD) to include zero. These thresholds align with ICH M10 bioanalytical validation guidelines and previous microsampling studies in oncology.

Statistical analyses were performed on paired samples where both capillary concentrations and corresponding average venous whole-blood concentrations (derived from triplicate measurements) were available. This yielded 23 paired observations from 13 patients, with 10 patients contributing measurements at two pharmacokinetically informative time points.

Bland–Altman analyses were conducted using both absolute differences (capillary − venous) and relative percent differences [100 × (capillary − venous)/venous], with mean bias and 95% limits of agreement defined as bias ± 1.96 standard deviations. The normality of paired differences was evaluated using the Shapiro–Wilk test, along with visual inspection of histograms and Q–Q plots. Proportional bias was further assessed by linear regression of the differences against the mean of the two methods. Passing–Bablok regression, a non-parametric method robust to outliers and distributional assumptions, was used to evaluate constant and proportional bias, with analytical confidence intervals for slope and intercept.

To quantify uncertainty in agreement estimates, non-parametric bootstrap resampling (5000 iterations) was applied to derive 95% confidence intervals for Bland–Altman bias and limits of agreement, as well as for the Passing–Bablok slope and intercept. Sensitivity to individual observations and repeated measurements was evaluated using leave-one-out and leave-one-patient-out analyses.

Independence of measurement errors was evaluated by inspection of residual plots for both Bland–Altman and Passing–Bablok analyses. No substantial deviations from these assumptions were observed.

Statistical analyses were performed using R version 4.5.2 for all regression, resampling, and assumption-checking procedures, while Microsoft Excel (Version 2511) and XLSTAT (XLSTAT 2025.1.3) were used for initial data processing and descriptive statistics.

Secondary endpoints included intra-method reproducibility, which was assessed for both Lab-TD and Traditional venous workflows, defined as an intra-assay coefficient of variation (CV) ≤ 15% across technical replicates. In addition, the operational robustness of the TD-EPI workflow was evaluated by assessing sample stability after 72 h of ambient storage.

All quantitative analysis was based on LC–MS/MS-derived peak-area ratios of epirubicin to the IS (doxorubicin), processed using Waters TargetLynx™ (4.0). Concentrations were calculated using calibration curves, and calibration linearity was confirmed using ordinary least-squares regression.

### 4.14. Safety Considerations

Epirubicin, doxorubicin, and daunorubicin are cytotoxic anthracycline anticancer agents and were handled with appropriate safety precautions. All preparation of stock solutions, calibrators, quality controls, and sample processing involving these compounds was performed in designated laboratory areas using certified chemical fume hoods or safety cabinets. Personnel wore suitable personal protective equipment, including laboratory coats, disposable nitrile gloves, and eye protection.

Contaminated consumables and chemical waste were collected and disposed of as cytotoxic hazardous waste in accordance with institutional safety procedures and local regulations.

## 5. Conclusions

This study provides method validation of the True Dose^®^ TD-EPI kit for decentralized TDM of epirubicin, demonstrating analytical equivalence to conventional venous methods under real-world conditions. By enabling accurate exposure assessment through capillary microsampling and ambient-temperature transport, the kit addresses key logistical barriers that have historically limited the clinical adoption of TDM.

These findings support its potential to improve chemotherapy safety, enable timely dose adjustments, and expand access to precision oncology, particularly for patients in rural or resource-limited settings. The TD platform also aligns with EU healthcare priorities, promoting equitable, decentralized, and sustainable cancer care.

Future research should focus on validating this approach across additional drug classes and tumor types, and on enabling fully patient-led sampling to support broader clinical integration.

## Figures and Tables

**Table 1:** Comparison of epirubicin concentrations from capillary microsampling (Cap-TD) and venous sampling processed either with the True Dose workflow (Lab-TD) or the standard reference method (Traditional) across two pharmacokinetic time points (C1S1: 2.5 h; C1S2: 48 h).

Patient	Epirubicin Cycle	Sample (Time Point)	Cap-TD/Lab-TD Ratio	Cap-TD/Traditional Ratio	Lab-TD CV% (Intra-Assay)	Traditional CV% (Intra-Assay)
P1	C1	S1 (2.5 h)	0.985	1.117	3.479	11.533
P1	C1	S2 (48 h)	0.720	1.131	1.432	2.428
P2	C1	S1 (2.5 h)	0.912	0.915	5.390	2.948
P2	C1	S2 (48 h)	0.842	1.120	2.941	7.132
P3	C1	S1 (2.5 h)	0.948	0.960	2.297	5.681
P3	C1	S2 (48 h)	NA **	NA **	NA **	NA **
P4	C1	S1 (2.5 h)	0.692 *	0.690 *	0.248	1.135
P4	C1	S2 (48 h)	0.870	1.504 °	1.396	3.985
P5	C1	S1 (2.5 h)	0.991	1.035	0.173	1.610
P5	C1	S2 (48 h)	0.703 ^	1.082	2.045	6.147
P6	C1	S1 (2.5 h)	1.021	1.016	6.363	0.419
P6	C1	S2 (48 h)	1.072	1.253	10.089	20.94
P7	C1	S1 (2.5 h)	0.961	1.083	1.889	1.360
P7	C1	S2 (48 h)	0.908	1.234	2.501	1.869
P8	C1	S1 (2.5 h)	1.021	1.013	9.399	2.989
P8	C1	S2 (48 h)	0.896	0.951	2.222	10.958
P9	C1	S1 (2.5 h)	0.889	0.959	8.133	0.899
P9	C1	S2 (48 h)	0.907	1.355	11.798	1.268
P10	C1	S1 (2.5 h)	1.033	1.121	2.131	1.260
P10	C1	S2 (48 h)	0.365 °	0.711 °	5.451	5.699
P11	C1	S1 (2.5 h)	0.810	1.392	8.392	2.354
P11	C1	S2 (48 h)	0.850	1.732	1.855	3.614
P12	C1	S1 (2.5 h)	0.945	1.373	4.343	1.408
P12	C1	S2 (48 h)	NA **	NA **	NA **	NA **
P13	C1	S1 (2.5 h)	1.054	1.305 ^§^	2.639	4.682
P13	C1	S2 (48 h)	NA **	NA **	NA **	NA **

Ratios reflect agreement between methods; values approaching 1.0 indicate concordance. Intra-assay precision for venous workflows is presented as the coefficient of variation (CV%) across triplicate replicates. Sample-specific deviations related to capillary collection are annotated. * Sample volume insufficient (<50 µL); ° Prolonged sampling; ^ Device issue (e.g., leakage); ^§^ Device-related error in Lab-TD replicate; ** Sample not collected due to unavailability of mobile nurse. Abbreviations: Cap-TD, capillary blood processed using the True Dose^®^ TD-EPI kit; Lab-TD, venous blood processed using the True Dose^®^ workflow; Traditional, standard venous LC–MS/MS preparation; C1, chemotherapy cycle 1; S1/S2, 2.5 h and 48 h post-infusion, respectively; CV, coefficient of variation; NA, Not Applicable.

**Table 2:** Key differences across capillary (Cap-TD) and venous (Lab-TD, Traditional) epirubicin sampling workflows.

Feature	Cap-TD (Capillary)	Lab-TD (Venous)	Traditional (Venous)
Sample Source	Fingerstick (capillary blood)	Venipuncture (venous blood)	Venipuncture (venous blood)
Collection Volume	50 µL	50 µL in triplicate	50 µL in triplicate
Internal Standard (IS)	Pre-loaded in the cap	Pre-loaded in the cap	Added manually in the lab
Stabilization Solvent	Pre-filled in a microtube cap	Pre-filled in a microtube cap	Added manually in the lab
Storage Conditions	Room temperature, 72 h	Room temperature, 72 h	−26 °C (frozen), 72 h
Processing	Manual shaking, followed by sonication and centrifugation at the lab	Manual shaking, followed by sonication and centrifugation at the lab	Thawing, vortexing, sonication, and centrifugation in the lab
Analytical Platform	LC–MS/MS	LC–MS/MS	LC–MS/MS
Intended Use	Decentralized/home sampling	Lab-based simulation of Cap-TD workflow	Conventional clinical lab method

Cap-TD, capillary-based True Dose^®^ workflow; Lab-TD, venous True Dose^®^ analog; Traditional, standard clinical reference workflow. Manual shaking was performed by the patient (Cap-TD) or lab personnel (Lab-TD); vortexing was performed mechanically (Traditional); Triplicate, three technical replicates per sample. Abbreviations: IS, internal standard; LC–MS/MS, liquid chromatography–tandem mass spectrometry.

## Data Availability

The datasets generated during the current study are not publicly available due to privacy and ethical restrictions, but are available from the corresponding author upon reasonable request.

## Supplementary Materials

The following supporting information can be downloaded at https://www.mdpi.com/article/10.3390/ph19020226/s1. Table S1. Epirubicin LC–MS/MS Quality Control (QC) Performance. Table S2. Data values of Epirubicin, Doxorubicin of Capillary (Cap-TD), Venous (Lab-TD), and the Traditional method. Figure S1. Leave-one-out sensitivity analysis for the Bland–Altman mean bias between capillary True Dose^®^ (Cap-TD) and traditional venous epirubicin measurements. Each point represents the recalculated mean bias after sequential exclusion of one paired observation. The dashed vertical line indicates the mean bias estimated using the full dataset. Figure S2. Leave-one-out sensitivity analysis for the Passing–Bablok regression slope comparing capillary True Dose^®^ (Cap-TD) and traditional venous epirubicin measurements. Each point represents the regression slope recalculated after sequential exclusion of one paired observation. The dashed vertical line indicates the slope estimated using the full dataset.
