# Supplementary material for: Method-Comparison Validation of a Novel Capillary Blood Collection Kit, True Dose® TD-EPI, for Therapeutic Drug Monitoring of Epirubicin
Source: Pharmaceuticals (Basel). 2026 Jan 28;19(2):226. doi: 10.3390/ph19020226 (PMC12943611; doi:10.3390/ph19020226)
Supplement: Supplementary file 1 [file pharmaceuticals-19-00226-s001.zip › pharmaceuticals-4087152-supplementary.pdf]

Supplementals

Tables S1 and S2.

Table S1. Epirubicin LC–MS/MS Quality Control (QC) Performance

QC Level	Nominal Conc. (µM)	Back-calculated Mean (µM)	Recovery %
QCL	0.060	0,058	96,38
QCM	0.600	0,608	101,33
QCH	1.500	1,474	98,25

Performance of the validated assay across five QC levels. All values met ICH M10 criteria for accuracy and precision.

Abbreviations: LQC: Low Quality Control; MQC: Medium Quality Control; HQC: High Quality Control; CV: Coefficient of Variation; µM: Micromolar; QC: Quality Control

Table S2. Data values of Epirubicin, Doxorubicin of Capillary (Cap-TD), Venous (Lab-TD), and the Traditional method.

Sample ID	Patient ID – sample type	EPI (AUC)	DOX (AUC)	EPI / DOX Ratio	EPI Concentration (µM)
ISO25-01_Clin_Samp_Run1_021	Patient 1 (S1) Cap-TD	5590	7260	0.77	0.229
ISO25-01_Clin_Samp_Run1_022	Patient 1 (S1) Lab-TD (1/3)	5260	6490	0.81	0.241
ISO25-01_Clin_Samp_Run1_023	Patient 1 (S1) Lab-TD (2/3)	5030	6660	0.755	0.225
ISO25-01_Clin_Samp_Run1_024	Patient 1 (S1) Lab-TD (3/3)	5750	7390	0.778	0.231
ISO25-01_Clin_Samp_Run1_025	Patient 1 (S1) Traditional (1/3)	4880	6250	0.781	0.232
ISO25-01_Clin_Samp_Run1_026	Patient 1 (S1) Traditional (2/3)	4860	7450	0.652	0.195
ISO25-01_Clin_Samp_Run1_027	Patient 1 (S1) Traditional (3/3)	1330	2110	0.629	0.188
ISO25-01_Clin_Samp_Run1_034	Patient 1 (S2) Cap-TD	1510	5910	0.255	0.077
ISO25-01_Clin_Samp_Run1_035	Patient 1 (S2) Lab-TD (1/3)	985	2770	0.356	0.107
ISO25-01_Clin_Samp_Run1_036	Patient 1 (S2) Lab-TD (2/3)	545	1560	0.349	0.105
ISO25-01_Clin_Samp_Run1_037	Patient 1 (S2) Lab-TD (3/3)	220	615	0.358	0.108
ISO25-01_Clin_Samp_Run1_038	Patient 1 (S2) Traditional (1/3)	1050	5590	0.189	0.057

ISO25-01_Clin_Samp_Run1_039	Patient 1 (S2) Traditional (2/3)	1050	5330	0.198	0.060
ISO25-01_Clin_Samp_Run1_040	Patient 1 (S2) Traditional (3/3)	1060	5460	0.195	0.059
ISO25-01_Clin_Samp_Run1_060	Patient 2 (S1) Cap-TD	3760	5550	0.678	0.202
ISO25-01_Clin_Samp_Run1_061	Patient 2 (S1) Lab-TD (1/3)	3120	4460	0.698	0.208
ISO25-01_Clin_Samp_Run1_062	Patient 2 (S1) Lab-TD (2/3)	3190	4110	0.776	0.231
ISO25-01_Clin_Samp_Run1_063	Patient 2 (S1) Lab-TD (3/3)	1990	2630	0.757	0.225
ISO25-01_Clin_Samp_Run1_064	Patient 2 (S1) Traditional (1/3)	5750	7540	0.763	0.227
ISO25-01_Clin_Samp_Run1_065	Patient 2 (S1) Traditional (2/3)	2320	3120	0.743	0.221
ISO25-01_Clin_Samp_Run1_066	Patient 2 (S1) Traditional (3/3)	2270	3170	0.719	0.214
ISO25-01_Clin_Samp_Run2_035	Patient 2 (S2) Cap-TD	628	4520	0.139	0.040
ISO25-01_Clin_Samp_Run2_036	Patient 2 (S2) Lab-TD (1/3)	775	4720	0.164	0.048
ISO25-01_Clin_Samp_Run2_037	Patient 2 (S2) Lab-TD (2/3)	738	4630	0.159	0.046
ISO25-01_Clin_Samp_Run2_038	Patient 2 (S2) Lab-TD (3/3)	745	4410	0.169	0.049
ISO25-01_Clin_Samp_Run2_039	Patient 2 (S2) Traditional (1/3)	529	4530	0.117	0.034

ISO25-01_Clin_Samp_Run2_040	Patient 2 (S2) Traditional (2/3)	517	3870	0.134	0.039
ISO25-01_Clin_Samp_Run2_041	Patient 2 (S2) Traditional (3/3)	530	4340	0.122	0.035
ISO25-01_Clin_Samp_Run1_073	Patient 3 (S1) Cap-TD	2980	4550	0.654	0.195
ISO25-01_Clin_Samp_Run1_074	Patient 3 (S1) Lab-TD (1/3)	2630	3850	0.682	0.204
ISO25-01_Clin_Samp_Run1_075	Patient 3 (S1) Lab-TD (2/3)	3100	4590	0.677	0.202
ISO25-01_Clin_Samp_Run1_076	Patient 3 (S1) Lab-TD (3/3)	3580	5070	0.707	0.211
ISO25-01_Clin_Samp_Run1_077	Patient 3 (S1) Traditional (1/3)	531	824	0.644	0.192
ISO25-01_Clin_Samp_Run1_078	Patient 3 (S1) Traditional (2/3)	1130	1570	0.723	0.215
ISO25-01_Clin_Samp_Run1_079	Patient 3 (S1) Traditional (3/3)	1480	2190	0.676	0.202
ISO25-01_Clin_Samp_Run2_022	Patient 4 (S1) Cap-TD	2040	3680	0.554	0.161
ISO25-01_Clin_Samp_Run2_023	Patient 4 (S1) Lab-TD (1/3)	2640	3270	0.806	0.233
ISO25-01_Clin_Samp_Run2_024	Patient 4 (S1) Lab-TD (2/3)	3280	4080	0.803	0.232
ISO25-01_Clin_Samp_Run2_025	Patient 4 (S1) Lab-TD (3/3)	1850	2310	0.801	0.232
ISO25-01_Clin_Samp_Run2_026	Patient 4 (S1) Traditional (1/3)	3730	4670	0.798	0.231

ISO25-01_Clin_Samp_Run2_027	Patient 4 (S1) Traditional (2/3)	3820	4680	0.816	0.236
ISO25-01_Clin_Samp_Run2_028	Patient 4 (S1) Traditional (3/3)	3830	4770	0.803	0.232
ISO25-01_Clin_Samp_Run2_048	Patient 4 (S2) Cap-TD	1430	3880	0.369	0.108
ISO25-01_Clin_Samp_Run2_049	Patient 4 (S2) Lab-TD (1/3)	1850	4320	0.429	0.125
ISO25-01_Clin_Samp_Run2_050	Patient 4 (S2) Lab-TD (2/3)	1970	4690	0.419	0.122
ISO25-01_Clin_Samp_Run2_051	Patient 4 (S2) Lab-TD (3/3)	1980	4630	0.428	0.125
ISO25-01_Clin_Samp_Run2_052	Patient 4 (S2) Traditional (1/3)	1030	4100	0.252	0.074
ISO25-01_Clin_Samp_Run2_053	Patient 4 (S2) Traditional (2/3)	608	2580	0.235	0.069
ISO25-01_Clin_Samp_Run2_054	Patient 4 (S2) Traditional (3/3)	1360	5420	0.251	0.073
ISO25-01_Clin_Samp_Run2_061	Patient 5 (S1) Cap-TD	2030	1770	1.15	0.330
ISO25-01_Clin_Samp_Run2_062	Patient 5 (S1) Lab-TD (1/3)	3390	2920	1.16	0.333
ISO25-01_Clin_Samp_Run2_063	Patient 5 (S1) Lab-TD (2/3)	4740	4080	1.16	0.332
ISO25-01_Clin_Samp_Run2_064	Patient 5 (S1) Lab-TD (3/3)	2910	2500	1.16	0.333
ISO25-01_Clin_Samp_Run2_065	Patient 5 (S1) Traditional (1/3)	402	360	1.12	0.320

ISO25-01_Clin_Samp_Run2_066	Patient 5 (S1) Traditional (2/3)	3280	3000	1.09	0.313
ISO25-01_Clin_Samp_Run2_067	Patient 5 (S1) Traditional (3/3)	2680	2380	1.13	0.323
ISO25-01_Clin_Samp_Run2_071	Patient 5 (S2) Cap-TD	1390	3860	0.361	0.105
ISO25-01_Clin_Samp_Run2_072	Patient 5 (S2) Lab-TD (1/3)	1820	3480	0.522	0.152
ISO25-01_Clin_Samp_Run2_073	Patient 5 (S2) Lab-TD (2/3)	2020	3920	0.515	0.150
ISO25-01_Clin_Samp_Run2_074	Patient 5 (S2) Lab-TD (3/3)	2010	4000	0.503	0.146
ISO25-01_Clin_Samp_Run2_075	Patient 5 (S2) Traditional (1/3)	425	1260	0.338	0.099
ISO25-01_Clin_Samp_Run2_076	Patient 5 (S2) Traditional (2/3)	244	788	0.31	0.090
ISO25-01_Clin_Samp_Run2_077	Patient 5 (S2) Traditional (3/3)	82.5	237	0.348	0.102
ISO25-01_Clin_Samp_Run2_087	Patient 6 (S1) Cap-TD	3780	3890	0.973	0.280
ISO25-01_Clin_Samp_Run2_088	Patient 6 (S1) Lab-TD (1/3)	40.9	46.4	0.882	0.254
ISO25-01_Clin_Samp_Run2_089	Patient 6 (S1) Lab-TD (2/3)	4170	4200	0.993	0.286
ISO25-01_Clin_Samp_Run2_090	Patient 6 (S1) Lab-TD (3/3)	4560	4660	0.979	0.282
ISO25-01_Clin_Samp_Run2_091	Patient 6 (S1) Traditional (1/3)	8470	8820	0.96	0.276

ISO25-01_Clin_Samp_Run2_092	Patient 6 (S1) Traditional (2/3)	8350	8700	0.959	0.276
ISO25-01_Clin_Samp_Run2_093	Patient 6 (S1) Traditional (3/3)	5860	6160	0.952	0.274
ISO25-01_Clin_Samp_Run2_100	Patient 6 (S2) Cap-TD	1090	3770	0.289	0.084
ISO25-01_Clin_Samp_Run2_101	Patient 6 (S2) Lab-TD (1/3)	1280	4290	0.298	0.087
ISO25-01_Clin_Samp_Run2_102	Patient 6 (S2) Lab-TD (2/3)	1080	4440	0.244	0.071
ISO25-01_Clin_Samp_Run2_103	Patient 6 (S2) Lab-TD (3/3)	1250	4670	0.267	0.078
ISO25-01_Clin_Samp_Run2_104	Patient 6 (S2) Traditional (1/3)	2190	8430	0.259	0.076
ISO25-01_Clin_Samp_Run2_105	Patient 6 (S2) Traditional (2/3)	2160	8400	0.257	0.075
ISO25-01_Clin_Samp_Run2_106	Patient 6 (S2) Traditional (3/3)	1420	8080	0.176	0.051
ISO25-01_Clin_Samp_Run2_127	Patient 7 (S1) Cap-TD	5610	6340	0.886	0.256
ISO25-01_Clin_Samp_Run2_128	Patient 7 (S1) Lab-TD (1/3)	6600	7010	0.942	0.271
ISO25-01_Clin_Samp_Run2_129	Patient 7 (S1) Lab-TD (2/3)	6290	6800	0.925	0.267
ISO25-01_Clin_Samp_Run2_130	Patient 7 (S1) Lab-TD (3/3)	6880	7600	0.906	0.261
ISO25-01_Clin_Samp_Run2_131	Patient 7 (S1) Traditional (1/3)	6450	7780	0.829	0.240

ISO25-01_Clin_Samp_Run2_132	Patient 7 (S1) Traditional (2/3)	5620	6950	0.809	0.234
ISO25-01_Clin_Samp_Run2_133	Patient 7 (S1) Traditional (3/3)	5420	6670	0.812	0.235
ISO25-01_Clin_Samp_Run2_140	Patient 7 (S2) Cap-TD	1460	7100	0.206	0.060
ISO25-01_Clin_Samp_Run2_141	Patient 7 (S2) Lab-TD (1/3)	1330	5730	0.232	0.068
ISO25-01_Clin_Samp_Run2_142	Patient 7 (S2) Lab-TD (2/3)	1310	5770	0.227	0.066
ISO25-01_Clin_Samp_Run2_143	Patient 7 (S2) Lab-TD (3/3)	1320	5990	0.221	0.064
ISO25-01_Clin_Samp_Run2_144	Patient 7 (S2) Traditional (1/3)	1070	6550	0.164	0.048
ISO25-01_Clin_Samp_Run2_145	Patient 7 (S2) Traditional (2/3)	1010	5990	0.168	0.049
ISO25-01_Clin_Samp_Run2_146	Patient 7 (S2) Traditional (3/3)	1090	6410	0.169	0.049
ISO25-01_Clin_Samp_Run2_153	Patient 8 (S1) Cap-TD	3530	5900	0.599	0.174
ISO25-01_Clin_Samp_Run2_154	Patient 8 (S1) Lab-TD (1/3)	3740	5830	0.641	0.186
ISO25-01_Clin_Samp_Run2_155	Patient 8 (S1) Lab-TD (2/3)	3930	6680	0.589	0.171
ISO25-01_Clin_Samp_Run2_156	Patient 8 (S1) Lab-TD (3/3)	3760	7110	0.529	0.154
ISO25-01_Clin_Samp_Run2_157	Patient 8 (S1) Traditional (1/3)	3770	6240	0.604	0.176

ISO25-01_Clin_Samp_Run2_158	Patient 8 (S1) Traditional (2/3)	3280	5740	0.571	0.166
ISO25-01_Clin_Samp_Run2_159	Patient 8 (S1) Traditional (3/3)	3520	5930	0.593	0.173
ISO25-01_Clin_Samp_Run2_166	Patient 8 (S2) Cap-TD	897	7130	0.126	0.036
ISO25-01_Clin_Samp_Run2_167	Patient 8 (S2) Lab-TD (1/3)	940	6710	0.14	0.041
ISO25-01_Clin_Samp_Run2_168	Patient 8 (S2) Lab-TD (2/3)	944	6880	0.137	0.040
ISO25-01_Clin_Samp_Run2_169	Patient 8 (S2) Lab-TD (3/3)	915	6390	0.143	0.041
ISO25-01_Clin_Samp_Run2_170	Patient 8 (S2) Traditional (1/3)	632	4910	0.129	0.037
ISO25-01_Clin_Samp_Run2_171	Patient 8 (S2) Traditional (2/3)	792	6610	0.12	0.035
ISO25-01_Clin_Samp_Run2_172	Patient 8 (S2) Traditional (3/3)	864	5860	0.148	0.043
ISO25-01_Clin_Samp_Run2_179	Patient 9 (S1) Cap-TD	5370	7020	0.765	0.222
ISO25-01_Clin_Samp_Run2_180	Patient 9 (S1) Lab-TD (1/3)	5870	6190	0.949	0.273
ISO25-01_Clin_Samp_Run2_181	Patient 9 (S1) Lab-TD (2/3)	4940	5960	0.829	0.240
ISO25-01_Clin_Samp_Run2_182	Patient 9 (S1) Lab-TD (3/3)	5270	6450	0.817	0.236
ISO25-01_Clin_Samp_Run2_183	Patient 9 (S1) Traditional (1/3)	5010	6210	0.807	0.233

ISO25-01_Clin_Samp_Run2_184	Patient 9 (S1) Traditional (2/3)	5280	6570	0.803	0.232
ISO25-01_Clin_Samp_Run2_185	Patient 9 (S1) Traditional (3/3)	4840	6120	0.792	0.229
ISO25-01_Clin_Samp_Run2_192	Patient 9 (S2) Cap-TD	1730	6760	0.256	0.075
ISO25-01_Clin_Samp_Run2_193	Patient 9 (S2) Lab-TD (1/3)	2070	6510	0.319	0.093
ISO25-01_Clin_Samp_Run2_194	Patient 9 (S2) Lab-TD (2/3)	1660	6540	0.253	0.074
ISO25-01_Clin_Samp_Run2_195	Patient 9 (S2) Lab-TD (3/3)	1820	6610	0.275	0.080
ISO25-01_Clin_Samp_Run2_196	Patient 9 (S2) Traditional (1/3)	1260	6690	0.189	0.055
ISO25-01_Clin_Samp_Run2_197	Patient 9 (S2) Traditional (2/3)	1190	6180	0.193	0.056
ISO25-01_Clin_Samp_Run2_198	Patient 9 (S2) Traditional (3/3)	1270	6740	0.188	0.055
ISO25-01_Clin_Samp_Run2_205	Patient 10 (S1) Cap-TD	4130	5390	0.768	0.224
ISO25-01_Clin_Samp_Run2_206	Patient 10 (S1) Lab-TD (1/3)	4200	5680	0.739	0.222
ISO25-01_Clin_Samp_Run2_207	Patient 10 (S1) Lab-TD (2/3)	3820	5170	0.739	0.214
ISO25-01_Clin_Samp_Run2_208	Patient 10 (S1) Lab-TD (3/3)	4220	6210	0.681	0.214
ISO25-01_Clin_Samp_Run2_209	Patient 10 (S1) Traditional (1/3)	4270	6140	0.695	0.197

ISO25-01_Clin_Samp_Run2_210	Patient 10 (S1) Traditional (2/3)	4110	5970	0.689	0.202
ISO25-01_Clin_Samp_Run2_225	Patient 10 (S1) Traditional (3/3)	4130	5390	0.768	0.200
ISO25-01_Clin_Samp_Run2_232	Patient 10 (S2) Cap-TD	1100	6340	0.173	0.050
ISO25-01_Clin_Samp_Run2_233	Patient 10 (S2) Lab-TD (1/3)	2080	4660	0.447	0.130
ISO25-01_Clin_Samp_Run2_234	Patient 10 (S2) Lab-TD (2/3)	2000	4010	0.499	0.145
ISO25-01_Clin_Samp_Run2_235	Patient 10 (S2) Lab-TD (3/3)	1970	4170	0.473	0.138
ISO25-01_Clin_Samp_Run2_236	Patient 10 (S2) Traditional (1/3)	1190	5050	0.236	0.069
ISO25-01_Clin_Samp_Run2_237	Patient 10 (S2) Traditional (2/3)	1390	5940	0.233	0.068
ISO25-01_Clin_Samp_Run2_238	Patient 10 (S2) Traditional (3/3)	1570	6100	0.258	0.075
ISO25-01_Clin_Samp_Run2_245	Patient 11 (S1) Cap-TD	4370	5290	0.826	0.239
ISO25-01_Clin_Samp_Run2_246	Patient 11 (S1) Lab-TD (1/3)	5680	5710	0.995	0.286
ISO25-01_Clin_Samp_Run2_247	Patient 11 (S1) Lab-TD (2/3)	5100	4520	1.13	0.323
ISO25-01_Clin_Samp_Run2_248	Patient 11 (S1) Lab-TD (3/3)	5330	5550	0.959	0.276
ISO25-01_Clin_Samp_Run2_249	Patient 11 (S1) Traditional (1/3)	3840	6540	0.588	0.171

ISO25-01_Clin_Samp_Run2_250	Patient 11 (S1) Traditional (2/3)	3570	6190	0.577	0.168
ISO25-01_Clin_Samp_Run2_251	Patient 11 (S1) Traditional (3/3)	3380	5600	0.605	0.176
ISO25-01_Clin_Samp_Run2_258	Patient 11 (S2) Cap-TD	2430	5060	0.48	0.140
ISO25-01_Clin_Samp_Run2_259	Patient 11 (S2) Lab-TD (1/3)	2500	4450	0.562	0.164
ISO25-01_Clin_Samp_Run2_260	Patient 11 (S2) Lab-TD (2/3)	2350	4080	0.576	0.168
ISO25-01_Clin_Samp_Run2_261	Patient 11 (S2) Lab-TD (3/3)	2400	4300	0.556	0.162
ISO25-01_Clin_Samp_Run2_262	Patient 11 (S2) Traditional (1/3)	1510	5480	0.276	0.080
ISO25-01_Clin_Samp_Run2_263	Patient 11 (S2) Traditional (2/3)	1170	4350	0.268	0.078
ISO25-01_Clin_Samp_Run2_264	Patient 11 (S2) Traditional (3/3)	1240	4320	0.288	0.084
ISO25-01_Clin_Samp_Run2_271	Patient 12 (S1) Cap-TD	2490	3710	0.671	0.195
ISO25-01_Clin_Samp_Run2_272	Patient 12 (S1) Lab-TD (1/3)	3230	4420	0.731	0.212
ISO25-01_Clin_Samp_Run2_273	Patient 12 (S1) Lab-TD (2/3)	3360	4990	0.674	0.196
ISO25-01_Clin_Samp_Run2_274	Patient 12 (S1) Lab-TD (3/3)	3170	4360	0.728	0.211
ISO25-01_Clin_Samp_Run2_275	Patient 12 (S1) Traditional (1/3)	1410	2850	0.493	0.144

ISO25-01_Clin_Samp_Run2_276	Patient 12 (S1) Traditional (2/3)	1050	2190	0.48	0.140
ISO25-01_Clin_Samp_Run2_277	Patient 12 (S1) Traditional (3/3)	1900	3890	0.488	0.142
ISO25-01_Clin_Samp_Run2_284	Patient 13 (S1) Cap-TD	2230	3210	0.693	0.201
ISO25-01_Clin_Samp_Run2_285	Patient 13 (S1) Lab-TD (1/3)	2280	3550	0.641	0.186
ISO25-01_Clin_Samp_Run2_286	Patient 13 (S1) Lab-TD (2/3)	2870	4240	0.677	0.196
ISO25-01_Clin_Samp_Run2_287	Patient 13 (S1) Lab-TD (3/3)	2510	3830	0.656	0.190
ISO25-01_Clin_Samp_Run2_288	Patient 13 (S1) Traditional (1/3)	2060	4050	0.508	0.148
ISO25-01_Clin_Samp_Run2_289	Patient 13 (S1) Traditional (2/3)	1400	2690	0.52	0.152
ISO25-01_Clin_Samp_Run2_290	Patient 13 (S1) Traditional (3/3)	1730	3120	0.555	0.162

Abbreviations: Epi = Epirubicin, Dox = Doxorubicin, Dauno = Daunorubicin, AUC = Area Under the concentration-time Curve, TD = True Dose, S1/S2 = Sample 1 (2.5h post-infusion), Sample 2 (48h post-infusion), Cap-TD = Capillary blood processed using the True Dose kit; Lab-TD = Venous blood processed using the True Dose kit; Traditional = Standard LC-MS/MS method using venous blood.

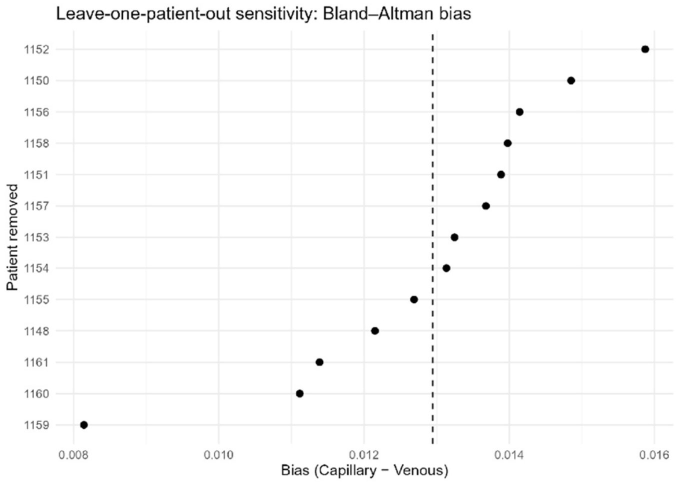

Figure S1. Leave-one-out sensitivity analysis for the Bland–Altman mean bias between capillary True Dose® (Cap-TD) and traditional venous epirubicin measurements. Each point represents the recalculated mean bias after sequential exclusion of one paired observation. The dashed vertical line indicates the mean bias estimated using the full dataset.

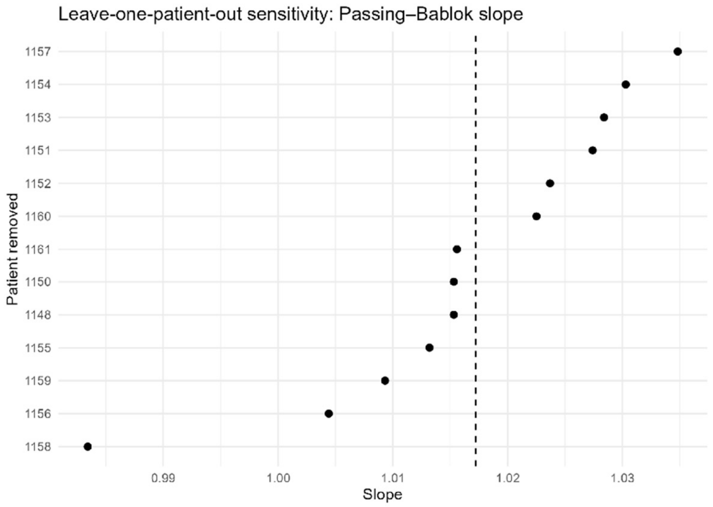

Figure S2. Leave-one-out sensitivity analysis for the Passing–Bablok regression slope comparing capillary True Dose® (Cap-TD) and traditional venous epirubicin measurements. Each point rep-resents the regression slope recalculated after sequential exclusion of one paired observation. The dashed vertical line indicates the slope estimated using the full dataset.
